# Update on Cardiovascular Risk and Obesity in Psoriatic Arthritis

**DOI:** 10.3389/fmed.2021.742713

**Published:** 2021-10-08

**Authors:** Julio Ramírez, Ana Belén Azuaga-Piñango, Raquel Celis, Juan D. Cañete

**Affiliations:** Arthritis Unit, Department of Rheumatology, Hospital Clínic, Barcelona, Spain

**Keywords:** psoriatic arthritis, comorbidities, obesity, cardiovascular risk, psoriasis

## Abstract

PsA is characterized by a high prevalence of cardiovascular (CV) comorbidities. Recognizing these comorbidities is critical due to their influence on the quality of life and the choice of therapy. Imaging techniques also play an important role in the evaluation of the CV risk in psoriatic disease, improving the prediction of CV events when combined with clinical scores as a predictive tool. Meta-analyses point to a significant reduction in the incidence of CV events associated with the suppression of inflammatory activity when using systemic therapies. Consequently, the mortality rate in PsA patients has fallen in the last 40 years and is now similar to that of the general population, including cardiovascular causes. Obesity is an especially relevant CV comorbidity in patients with psoriatic disease, most of whom are overweight/obese. Body mass index (BMI) is a risk factor for PsA and a causal relationship with psoriasis has been demonstrated by Mendelian randomized studies. The study of fat distribution shows that patients with psoriasis are characterized by visceral fat accumulation, which correlates with CV risk measurements. These findings suggest that approaches to the prevention and treatment of psoriatic disease might come from targeting adiposity levels, in addition to the immune pathways. Weight loss treatment with low energy diets in patients with PsA has been associated with significant improvements in disease activity. Novel strategies using a multimorbidity approach, focused more on patients outcomes, are necessary to better address comorbidities, improve clinical outcomes and the quality of life of patients with psoriatic disease.

## Introduction

Psoriatic arthritis (PsA) is one of the most common chronic inflammatory conditions, with a prevalence of 0.3–1% in the general population (1). PsA affects up to 30% of patients with psoriasis and leads to severe physical limitations and disability (2). In addition to skin and joint involvement, PsA is characterized by a high prevalence of comorbidities. More than half of PsA patients have ≥1 comorbidity (3), which have a significant negative impact on the quality of life. Recognizing and addressing comorbidities are critical to safely and effectively treating PsA patients as they often have implications not only for physical function and the quality of life but also the choice of therapy. For instance, obesity, hypertension, and a Charlson comorbidity index >1 are prognostic factors for worse treatment outcomes (4).

**Figure 1:**
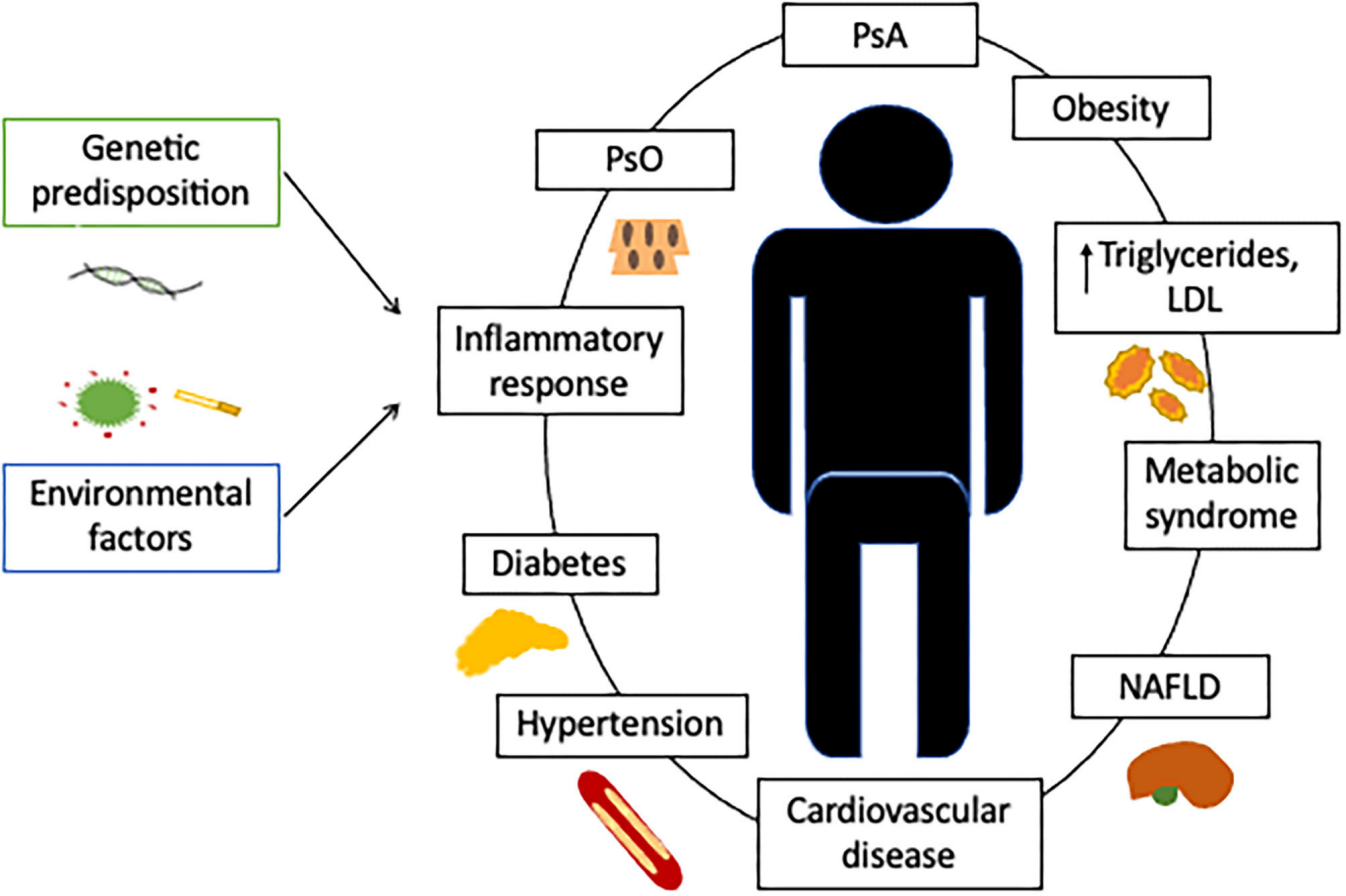
Cardiovascular comorbidities in patients with psoriatic arthritis. Although PsA can be associated with a higher prevalence of cardiovascular risk factors, these conditions do not fully account for the greater incidence of cardiovascular events. Associated factors, such as chronic systemic inflammation, a predisposing genetic background, or the baseline treatment, must contribute to the higher cardiovascular risk. Comorbidity is defined as the existence of any additional entity during the clinical course of a patient who has the index disease under study, such as PsA. In the comorbidity concept, the management and treatment are primarily focused on the index disease and the effect quantified by evaluating the disease activity. In the multimorbidity concept, the patient is of central concern and all coexisting diseases are of equal importance with interactions between each other. In the multimorbidity concept, the management and treatment focus on the patient and effectiveness is quantified by overall indicators such as quality of life or physical function (5). This new approach compels us to tackle with PsA patients from a multidisciplinary perspective. PsO, psoriasis; PsA, psoriathic arthritis; LDL, Low-density lipoprotein; NAFLD, Nonalcoholic fatty liver disease.

Despite advances in PsA therapy over the past 20 years, current outcomes are far from those achieved in psoriasis. The traditional approach to comorbidities is a part of the problem, as they are not considered in disease activity indexes, despite influencing inflammatory parameters such as C-reactive protein (CRP) and subjective scores (pain and general assessment). In contrast, the multimorbidity approach treats the patient as the central concern and all coexisting diseases and their interactions are of equal importance. In this model, management and treatment are focused on the patient and effectiveness is quantified by overall indicators such as the quality of life and physical function (5) (Figure 1).

## Prevalence of Cardiovascular Comorbidities in Psoriatic Arthritis

The prevalence of comorbidities associated with cardiovascular (CV) risk, such as hypertension or hyperlipidemia in PsA, varies geographically. Extensive data from American cohorts show that almost half of PsA patients have hypertension or hyperlipidemia and up to 20% have diabetes mellitus, while the prevalence of chronic ischemic heart disease is >11% (6). The rate of comorbidities, especially those related to CV risk, are lower in European countries, as recently shown in a Mediterranean cohort, where the prevalence of hypertension, hyperlipidemia and chronic ischemic heart disease were 39, 19.1, and 5.5%, respectively (7), suggesting marked geographic differences. A diverse genetic background and different diets are hypothetical explanations. Additional data from retrospective Taiwanese cohorts found an association between psoriasis and cerebrovascular disease [Hazard Ratio (HR) 1.27 (95% CI 1.05-1.52) for ischemic stroke (8) and HR 1.28 (95% CI 1.16–1.41)] for general cerebrovascular disease (9). Moreover, a cross-sectional study from Japan found an association with coronary heart disease [Odds ratio (OR) 1.27 (95% CI 1.01–1.58)] in patients with psoriasis (1197) vs. Hospital-based population (113,065) (10), demonstrating a higher CV risk also in Asian population.

## Cardiovascular Comorbidities, Hospitalization, and Mortality in Psoriatic Arthritis

Studies on all-cause mortality revealed mixed results, in part due to differences in PsA definition, patient population, disease duration, study design and therapy. In general, earlier cohorts showed an increased mortality compared with more recent studies (11, 12). In a Canadian PsA cohort with nearly 40 years of follow-up, the major causes of death included malignant neoplasms and acute myocardial infarction, but no disease was above the rate in the general population (13). A longitudinal cohort study performed in the United Kingdom evaluated the cause-specific mortality in patients with PsA compared with the general population and RA patients, finding that suicide (HR 3.03), but not CV (HR 1.09, 95% CI 0.91–1.32) deaths were elevated in PsA patients (14). In contrast, the results of another British study cohort of severe PsA receiving tumor necrosis factor inhibitors (TNFi) from 2002 to 2012 showed that all-cause mortality was increased (Standardized Mortality Ratio [SMR] 1.56; 95% CI 1.12–2.11). Death from malignancy did not increase, but death from coronary heart disease was higher than in the general population (SMR 2.42; 95% CI: 1.11–4.59) (15).

A retrospective US-based claims study with nearly 15,000 PsA patients and 35,037 matched controls found that PsA patients had higher incidence rates of CV disorders (hypertension, hyperlipidemia, coronary artery disease, cerebrovascular disease and peripheral vascular disease) and a higher rate of hospitalization due to CV disease than controls (general CV diagnosis: 14.4 vs. 9.4%, *p* < 0.05; coronary disease as primary diagnosis: 0.8 vs. 0.5%, *p* < 0.001) (16), although mortality rates were not analyzed.

## Subclinical Atherosclerosis in Psoriatic Arthritis

In addition to a higher incidence of CV risk factors, up to half of PsA patients have imaging evidence of atherosclerosis without traditional CV risk factors (12, 17). The relationship between subclinical atherosclerosis and PsA is complex, and traditional risk factors may not entirely explain the accelerated atherosclerotic process in these patients. Other mechanisms (i.e., inflammatory and immunological) have been proposed to explain the relationship between PsA and atherosclerosis. Chronic inflammation, which accelerates the atherosclerotic process, is believed to contribute to this increased risk (18, 19). Accordingly, suppression of inflammatory activity using treat-to-target strategies has a protective effect against plaque progression and atherosclerosis, as has been shown in rheumatoid arthritis (RA), psoriasis and PsA studies (20). In a recent study in 101 patients with PsA, achieving sustained minimal disease activity had a protective effect against plaque progression, as evaluated with carotid ultrasound, a finding independent of biologic disease-modifying anti-rheumatic drugs (bDMARDs) use, suggesting that controlling disease activity may be useful in improving the CV risk in these patients (21). Accelerated coronary plaque formation in PsA patients, particularly mixed plaques, was found on 64-slice coronary CT angiography. This accelerated process was independent of metabolic disease, suggesting disease activity and PsA severity may predict the burden of coronary plaque better than traditional risk factors (22). Taken together, imaging techniques play an important role in the evaluation of CV risk in psoriatic disease. The burden of carotid atherosclerosis, as estimated by carotid ultrasound, can improve the prediction of CV events, when combined with the Framingham risk score as a predictive tool (23).

## Global Cardiovascular Risk in Immuno-Mediated Diseases

It is known that RA patients have a higher incidence of major cardiovascular events (MACE) and a higher mortality rate than the general population. However, it is not clear whether the CV risk is also higher in psoriatic disease. The prevalence of traditional CV risk factors is higher in psoriatic disease but it is unclear whether this leads to excess mortality and whether PsA should be considered an independent risk factor for CV events such as RA or systemic lupus erythematosus (24, 25).

A British population-based study of MACE in immune-mediated diseases identified psoriatic disease as an independent risk factor for MACE, including myocardial infarction and stroke, although this was only significant in psoriasis and PsA patients not prescribed a disease-modifying anti-rheumatic drug (DMARD). The odds of MACE in RA patients were 39 and 58% higher than in the general population in DMARD and non-DMARD-treated RA patients, respectively (26).

**Figure 2:**
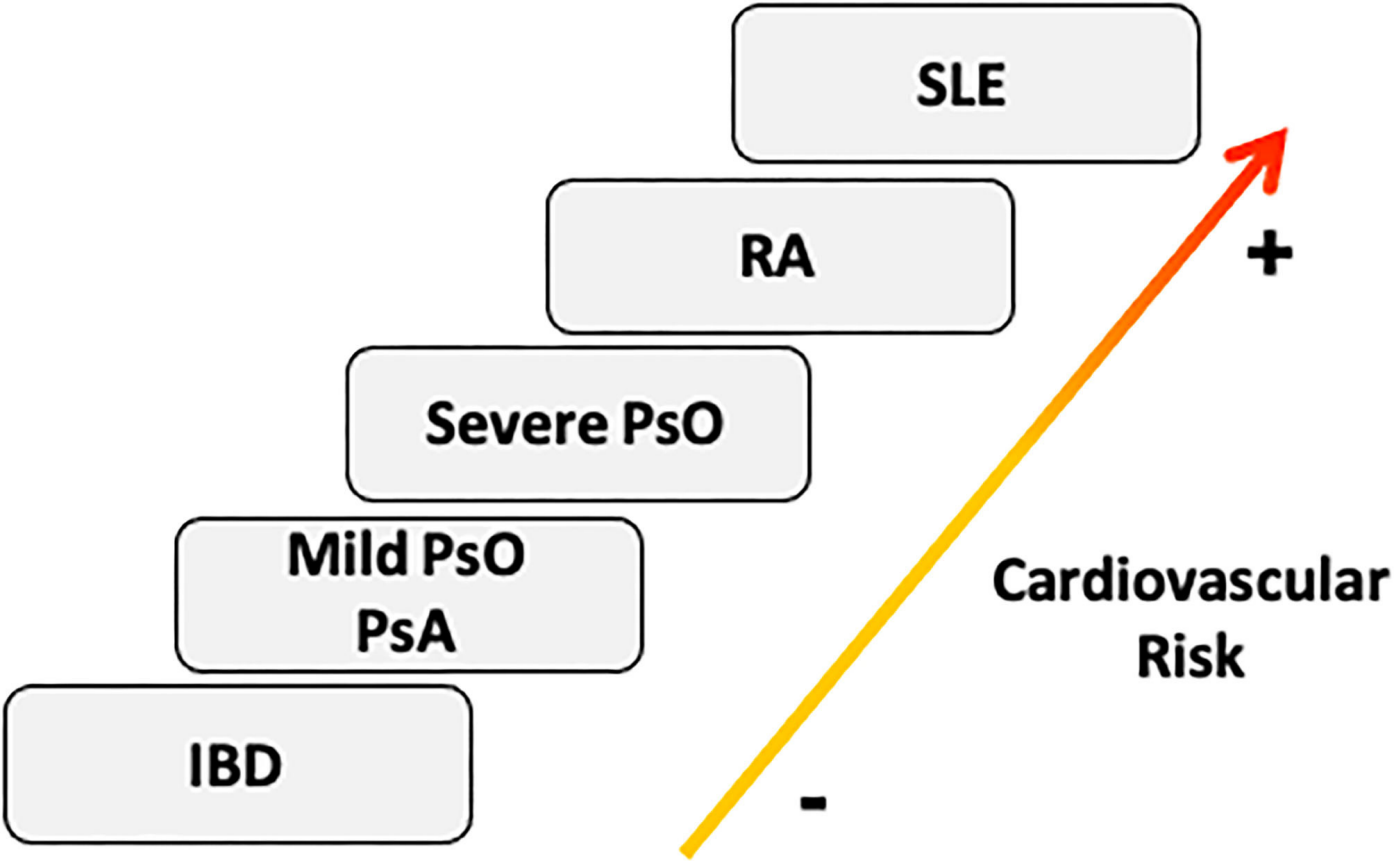
Cardiovascular risk stratification in Immunomediated diseases. According to the prevalence of cardiovascular risk factors and the incidence of cardiovascular events, we should consider SLE as the IMID with highest cardiovascular risk burden, followed by RA, PsO, PsA, and IBD (20). The severity of skin involvement could be essential to classify the cardiovascular profile of psoriatic disease (21). IBD, inflammatory bowel disease; PsA, psoriatic Arthritis; PsO, Psoriasis; RA, Rheumatoid Arthritis; SLE, systemic lupus erythematosus.

Taking all the evidence into account, RA should be included in the SCORE scale as an independent factor for CV events. Psoriatic disease should be considered as having the same risk as RA, especially psoriatic disease with severe skin involvement (26). PsA, mild skin psoriasis and inflammatory bowel disease should be probably placed on a lower level, with a hypothetically-lower risk of CV events (24, 25) (Figure 2), although the evidence is not clear on this point.

## Cardiovascular Risk Modification With Systemic Therapies in Psoriatic Disease

Meta-analyses show a significant reduction in the incidence of CV events associated with suppression of inflammatory activity using conventional DMARDs (RR 0.72, 95% CI 0.57–0.91 for methotrexate) or TNFi (RR 0.70, 95%CI 0.54–0.90) in PsA (27). The use of TNFi in RA was shown to reduce the risk of MACE over 8 years (28). Although there is increasing evidence that TNFi may be associated with a reduced risk of CV disease in patients with PsA, data on other biologic treatments are largely lacking.

A large study of 60,028 patients with psoriasis or PsA found no overall differential risk of incident atrial fibrillation and a composite CV endpoint of MI, stroke, and coronary revascularization associated with the use of ustekinumab (interleukin [IL]-12/IL-23 inhibitor) vs. TNFi (29). Ustekinumab has been shown to reduce systemic and vascular inflammation measured using ^18^F-fluorodeoxyglucose positron emission tomography-computed tomography (^18^F-FDG PET/CT) in patients with moderate to severe psoriasis achieving a PASI 75 response (30).

Given the efficacy of secukinumab and other anti-IL17 agents on the skin and musculoskeletal manifestations of psoriatic disease (31–34) and the lack of data on the effect of anti-IL17 on CV risk markers in psoriasis, the CARIMA (Evaluation of cardiovascular risk markers in psoriatic patients treated with secukinumab) study was designed to explore the effects of secukinumab on CV risk markers in patients with psoriasis. Flow-mediated dilation (FMD), a measure of endothelium-dependent control of vascular tone, was assessed as a parameter of vascular endothelial function and an early predictor of the CV prognosis. After one year of therapy, there was a 2% (p=0.002) improvement in FMD with 300 mg of secukinumab with no proatherogenic vessel wall changes or alterations in CV markers, indicating that IL17 inhibition might have a beneficial effect on the CV risk by improving the endothelial function of patients with plaque psoriasis (35). Whether this protective effect might also be seen in PsA remains unclear.

## Increased Weight/Body Mass Index and Obesity in Psoriatic Arthritis

Obesity is a major health problem worldwide and one of the biggest public health challenges to emerge in recent decades (36). A high proportion of patients with PsA are overweight (BMI >25) or obese (BMI>30) (37). An analysis of the CORRONA (Consortium of Rheumatology Researchers of North America) database found patients with PsA were a mean of 7.7 Kg heavier than patients with RA. Comparing the BMI in PsA (*n* = 5 644), psoriasis (*n* = 5 448), RA (*n* = 5 350), and the general population, the percentages with obesity were 37, 29, 27, and 18% respectively and the odds of obesity were 61% higher for patients with PsA (38).

Obesity is an independent factor for not achieving a therapeutic response in patients with psoriasis and PsA. A reduction in the clinical response has been found, especially for TNFi therapy, as shown by several studies and a recent meta-analysis (39), which found the odds of failing with TNFi therapy were almost two-fold higher for both psoriasis and PsA patients with obesity.

## Obesity as A Risk Factor for Psoriasis and Psoriatic Arthritis

Association between higher BMI and psoriasis has been shown by many observational studies (40). Recently, mendelian randomized analyses have provided evidence that a higher BMI increases the odds of psoriasis by 9% per 1 unit increase in BMI, but not the other way around (41). This implies that excess adiposity is part of the reason for some individuals developing psoriasis. Leptin can increase keratinocyte proliferation and proinflammatory protein secretion, which are characteristic of psoriasis (42), while the secretion of adiponectin, which is putatively anti-inflammatory (43), is reduced in obese persons. The skin of obese individuals shows features of impaired barrier function (44), while impairment in lymphatic function may delay the clearance of inflammatory mediators (45). Although further detailed study is required, these findings suggest that approaches to the prevention and treatment of psoriasis might include targeting adiposity levels, in addition to immune pathways in the skin. Although these results imply that such interventions may be effective in the prevention of psoriasis, it has not be determined whether would be effective in improving the disease course after onset.

Obesity could also be a key factor in the transition from skin psoriasis to PsA. Several studies suggest obesity is a risk factor for both psoriasis and PsA. A cohort study by Love et al., which was conducted using an electronic database of medical records representative of the general UK population, with a 15-year time horizon, found the incidence rates of PsA increased in tandem with BMI, both in the 75,395 people with psoriasis and in the general population (almost 2 million) (46). Li et al. analyzed information on BMI, weight change and measures of central obesity in participants in the US Nurse Health Study II (89,049 women) with a 14-year time horizon and found that BMI was monotonically associated with an increased risk of incident PsA. Moreover, there was a graded positive association between weight change from 18 years of age onwards and measures of central obesity, and the risk of PsA. A similar association was found in participants developing psoriasis during the follow-up (47). These studies offer valuable new information on the link between obesity and PsA and provide a potential opportunity to reduce the occurrence of PsA by encouraging a reduction in weight, a modifiable risk factor (48).

## Fat Mass Distribution in Psoriatic Disease

Another important issue is the way that fat mass is distributed in the body. Studies on adiposity in PsA and psoriasis generally refer to anthropometric measurements such as BMI, but this does not accurately reflect the visceral fat mass. Using dual energy X-ray absorptiometry (DXA), Toussirot et al. studied body composition and fat distribution (android and visceral fat) in patients with psoriasis and PsA. They found that patients with psoriasis are characterized by visceral fat accumulation, whereas the amount of fat in this region did not differ between PsA patients and controls. Furthermore, visceral adiposity in psoriasis correlated with CV risk measurements, such as SCORE (49).

Magnetic resonance imaging (MRI) may be the most accurate method of measuring the body composition. On MRI, PsA patients showed significantly greater visceral adipose tissue volume and liver fat percentage compared with matched metabolic disease-free controls, whereas the thigh muscle volume was lower. The authors concluded that body fat distribution in PsA is more in keeping with the pattern observed in type 2 diabetes and is more closely associated with cardiometabolic disease (50). These data support the need for a greater emphasis on weight loss in PsA management.

## Weight Loss Interventions as Part of Therapeutic Strategies in Psoriatic Arthritis

The concept of losing weight as an effective measure to improve outcomes in PsA has recently been tested. In 41 patients with PsA and obesity, weight loss treatment with very-low energy diets (640 Kcal/day for 12–16 weeks, followed by a structured reintroduction of an energy-restricted diet) resulted in a median weight loss of 18.6% and was associated with significant improvement in disease activity in the joints, entheses and skin at 6 months. Greater weight loss resulted in improvements in a dose-response manner. The treatment was effective, safe and well tolerated. In addition, an association between higher BMI and increased disease activity at baseline was demonstrated (51). After two years follow up, some PsA patients regained weight, but disease activity outcomes were maintained, and the number of patients with minimal disease activity increased from 28.2% at baseline to 45.7% at 24 months. The weight loss was also associated with improved levels of serum lipids, glucose and urate and antihypertensive treatment was reduced or stopped in several patients during the follow up (52). These results support the findings of previous studies showing better responses to TNFi and greater odds of achieving minimal disease activity after a 5% weight loss (53). Taken together, it seems that active weight loss strategies could be a choice in every PsA patient with overweight/obesity.

Whereas, TNFi are less effective in obese patients, new therapeutic options, such as ustekinumab 90 mg, seem to achieve the same clinical response regardless of the patient's weight, as shown in the *post-hoc* analysis of the PSUMMIT trials in PsA (54). Similarly, secukinumab seems efficacious irrespective of body weight in psoriasis clinical trials, especially at a dose of 300 mg (55). Pooled analysis of clinical trials of tofacitinib in PsA show higher efficacy than placebo at month 3 across all baseline BMI categories. However, like TNFi, reduced efficacy was generally observed in tofacitinib-treated and placebo-treated patients with baseline BMI > 35 compared with patients in the other baseline BMI categories (56).

The reason why TNFi and JAK inhibitors have reduced effectiveness in patients with obesity compared with other drugs are unclear; however, pharmacokinetic properties, the volume of distribution and lipophilicity may be contributing factors (57).

Accordingly, current guidelines for the treatment of PsA recommend weight loss in overweight and obese patients to potentially improve pharmacologic responses (58, 59).

## Discussion

Psoriasis and PsA are strongly associated with obesity and CV risk factors. Obesity increases the risk of psoriasis and PsA and is associated with greater disease activity, poorer treatment response and a lower chance of achieving minimal disease activity. Patients with PsA also have an increased risk of CV disease. Chronic inflammation, which accelerates the atherosclerotic process, in combination with a higher prevalence of CV risk factors is believed to contribute to this increased risk. Direct interventions with systemic therapies decrease inflammatory activity and potentially reduce the incidence of CV events.

There is an urgent need to improve the primary and secondary prevention of CV disease in patients with psoriasis and PsA. Lifestyle changes should be actively encouraged; risk stratification should be adjusted in patients with psoriasis and PsA; and correct pharmaceutical interventions should be introduced, and their effectiveness monitored. Physicians caring for patients with psoriasis and/or PsA should play an active role in achieving these goals in collaboration with general practitioners and cardiologists.

## Author Contributions

All authors listed have made a substantial, direct and intellectual contribution to the work, and approved it for publication.

## Conflict of Interest

The authors declare that the research was conducted in the absence of any commercial or financial relationships that could be construed as a potential conflict of interest.

## Publisher's Note

All claims expressed in this article are solely those of the authors and do not necessarily represent those of their affiliated organizations, or those of the publisher, the editors and the reviewers. Any product that may be evaluated in this article, or claim that may be made by its manufacturer, is not guaranteed or endorsed by the publisher.

## Abbreviations

bDMARDs: biological disease-modifying antirheumatic drugs
BMI: body mass index
CRP: C-recative protein
CT: computerized tomography
CV: cardiovascular
DMARDs: disease-modifying antirheumatic drugs
DXA: dual energy X-ray absorptiometry
IL: interleukin
MACE: major adverse cardiovascular events
MRI: magnetic resonance imaging
PsA: psoriatic Arthritis
RA: rheumatoid arthritis
SMR: standardized mortality ratio
TNFi: tumor necrosis factor-alpha inhibitor.
